# The role of branch architecture in assimilate production and partitioning: the example of apple (*Malus domestica*)

**DOI:** 10.3389/fpls.2014.00338

**Published:** 2014-07-09

**Authors:** Julienne Fanwoua, Emna Bairam, Mickael Delaire, Gerhard Buck-Sorlin

**Affiliations:** UMR 1345 Institut de Recherche en Horticulture et Semences, AGROCAMPUS OUEST-Centre d’Angers, AngersFrance

**Keywords:** apple, branch architecture, source/sink ratio, assimilate partitioning, vascular architecture, modeling

## Abstract

Understanding the role of branch architecture in carbon production and allocation is essential to gain more insight into the complex process of assimilate partitioning in fruit trees. This mini review reports on the current knowledge of the role of branch architecture in carbohydrate production and partitioning in apple. The first-order carrier branch of apple illustrates the complexity of branch structure emerging from bud activity events and encountered in many fruit trees. Branch architecture influences carbon production by determining leaf exposure to light and by affecting leaf internal characteristics related to leaf photosynthetic capacity. The dynamics of assimilate partitioning between branch organs depends on the stage of development of sources and sinks. The sink strength of various branch organs and their relative positioning on the branch also affect partitioning. Vascular connections between branch organs determine major pathways for branch assimilate transport. We propose directions for employing a modeling approach to further elucidate the role of branch architecture on assimilate partitioning.

## THE ROLE OF BRANCH ORGAN NUMBER AND POSITIONING ON ASSIMILATE PRODUCTION AND PARTITIONING

**FIGURE 1:**
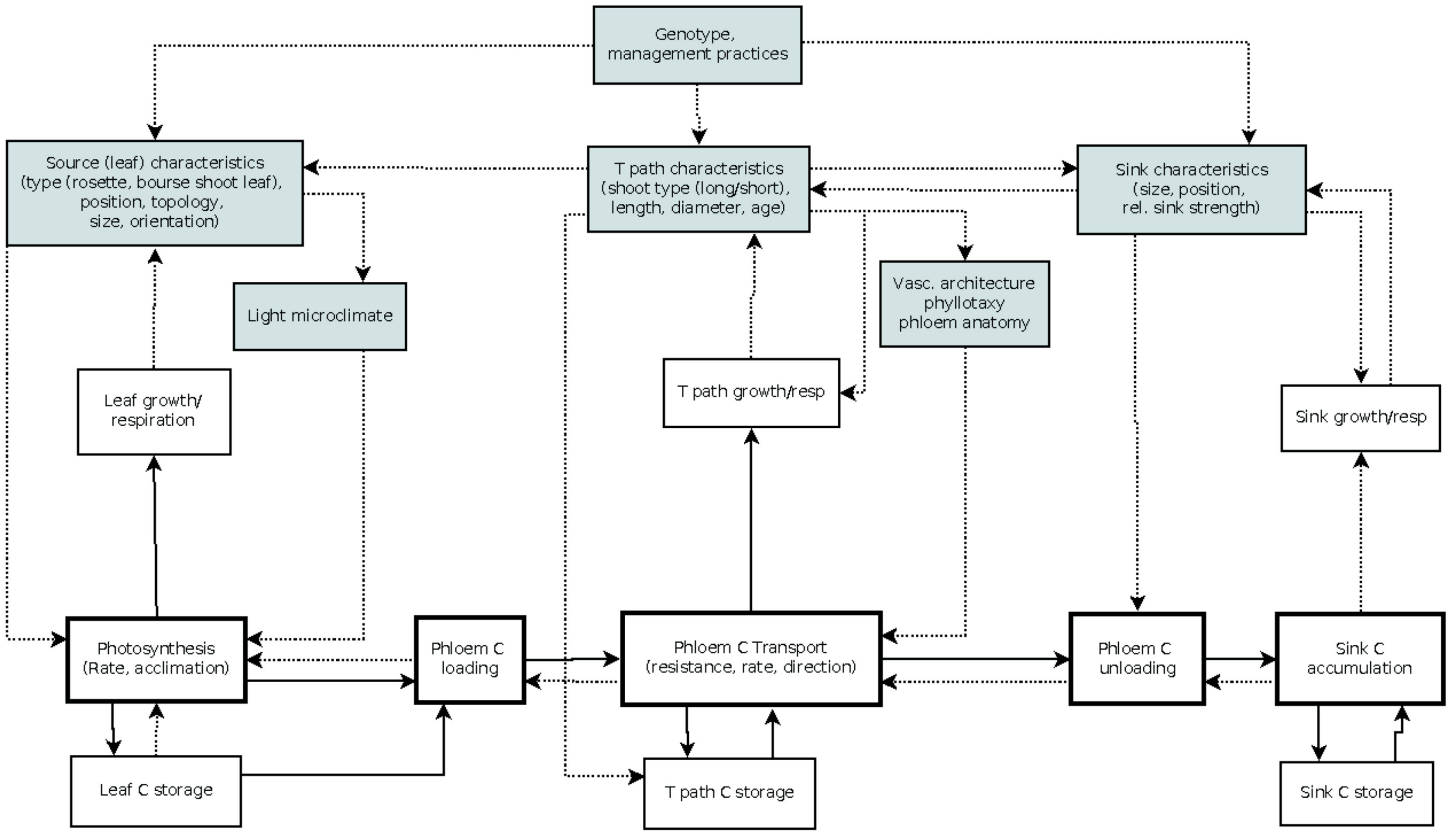
**Schematic representation of carbon assimilation, transport and allocation processes and branch architectural factors influencing them (gray shaded boxes).** Arrows indicate flow of carbon (plain line) or an influence on the process pointed at (dotted line); C, carbon; T path, transport path; rel, relative. For more information see text.

The first-order branch of an apple tree is the main structural component of the tree crown and the site of fruit production, comprising both source and sink structures. Botanically, it represents a succession of annual shoots resulting from the activity of vegetative and mixed buds located at different positions in the branch. Vegetative buds develop into vegetative annual shoots consisting of successions of metamers each of which is constituted of a node, a leaf, an internode, and an axillary bud (Room et al., 1994; Seleznyova et al., 2003). A mixed bud develops into a terminal inflorescence consisting of 5–7 distal flowers situated above a variable number of proximal preformed rosette leaves, a swollen axis called “bourse” carrying one or two axillary shoots (bourse shoots) developing from lateral sylleptic buds in the axils of the rosette leaves (Costes, 2003). Depending on the cultivar and environmental conditions, a proportion of buds resume their activity in spring resulting in the extension of new shoots (Lauri et al., 1996). Shoot extension results in short or long shoots depending on environmental conditions and position within the branch (Costes et al., 2006; Stephan et al., 2007). Each branch carries source and sink organs of which number and position within the branch affect the pattern of assimilate partitioning. An overview of the main processes characterizing carbon assimilation, transport, and allocation is given in **Figure 1**.

### ASSIMILATE PRODUCTION

The importance of leaf structure (shape, surface, and orientation) for assimilate production is generally associated with the effect of light interception on leaf photosynthesis (Tustin et al., 1992; Mierowska et al., 2002; Li and Lakso, 2004). Leaf orientation affects exposure to light and, consequently, photosynthesis rate. In trees with dense canopies the photosynthesis rate of shaded rosette and bourse shoot leaves located in the interior of the canopy is low, which negatively affects fruit yield (Wünsche and Lakso, 2000a). Variations in leaf photosynthesis within the apple branch may be linked to differences in leaf size, structure and function associated with shoot types or the proximity of major sink organs (Palmer, 1992; Schechter et al., 1992; Wünsche and Lakso, 2000b; Lauri and Kelner, 2001). Schechter et al. (1992) showed that leaf characteristics such as leaf weight, leaf internal gas content, chlorophyll content and water content differ between vegetative shoots and spurs. In the same study the presence or absence of fruits on a spur affected leaf characteristics. Carbon may also be supplied from reserves stored in branch structures. This carbon is especially important for bud break, initial shoot growth (Hansen, 1971) and for buffering deficit in leaf carbon supply (De Schepper et al., 2013a). The mechanisms of carbon storage/remobilization, their regulation and seasonal patterns in trees have been recently reviewed by Dietze et al. (2014).

Common branch manipulation practices such as pruning, branch girdling and bending may affect leaf photosynthesis via changes in leaf exposure to sunlight (Mierowska et al., 2002; Li and Lakso, 2004), variations in source-sink ratio or leaf characteristics (Blanke, 1997; Cheng et al., 2008). Summer pruning improves light penetration inside the canopy, re-exposes previously shaded leaves and improves their photosynthesis (Mierowska et al., 2002). However, the photosynthetic acclimation of previously shaded apple leaves might be limited by the pre-pruning light environment and the intensity of their re-exposure to light post-pruning (Li and Lakso, 2004). Leaf photosynthesis is inhibited following a reduction in fruit load, suggesting a sink limitation of photosynthesis (Palmer et al., 1997). Fruit load-induced inhibition in leaf photosynthesis has been mainly associated with a reduction in stomatal conductance or an increase in leaf starch content (Palmer, 1992; Blanke, 1997; Wünsche et al., 2005). Girdling treatment blocks export of branch assimilates to other tree parts and may induce conditions of sink limitation and inhibition of photosynthesis (Cheng et al., 2008; Fan et al., 2010). The reduction of leaf photosynthesis on girdled branches has been associated with a closure of leaf stomata, a reduction in the activity of ribulose-1,5-bisphosphate carboxylase and an increase in leaf starch content in apple and other fruit tree species (Davie et al., 1995; Cheng et al., 2008; Fan et al., 2010). Bending is commonly used in the orchard to inhibit shoot growth and promote flowering (Han et al., 2007). It is thought to have a gravimorphic effect on the bent branch, inducing a reduction in apical dominance (Wilson, 2000). Han et al. (2008) observed that increasing apple branch bending angle from 55 to 110^∘^ increased leaf thickness, leaf stomatal conductance, and leaf photosynthesis rate.

### ASSIMILATE PARTITIONING

Carbon partitioning involves transport of assimilates from source organs and their distribution to various sinks. Sources and sinks are connected to each other via conducting and supporting shoot structures constituting the architecture of the branch. The influence of this architecture on assimilate partitioning is complex and not yet well understood. Experiments with radioactive carbon provide some insights (Hansen, 1967a; Tustin et al., 1992; Grappadelli et al., 1994), especially into the role of the developmental stage and position of a source or sink.

In the early season, shoot and fruit growth occur simultaneously on the apple branch leading to competition between vegetative and generative sinks. In the first 2 weeks after bloom young apple fruits are almost entirely supplied by rosette leaves (Tustin et al., 1992). During this period, assimilates produced by bourse shoot and extension shoot leaves are exported to their shoot tips and young leaves (Tustin et al., 1992; Grappadelli et al., 1994). Shoot tips and young leaves are stronger sinks for assimilates than flowers and young fruits. Grappadelli et al. (1994) estimated that young apple leaves are net sinks. Johnson and Lakso (1986) indicated that, at the shoot scale, assimilate transport out of the shoot does not occur until a minimum number of unfolded leaves is attained. From 3 to 5 weeks after bloom, up to 80 and 50% of carbon, respectively, fixed by rosette and bourse shoot leaves are directed to the fruit (Tustin et al., 1992; Wünsche and Lakso, 2000a). Early fruit growth may also rely on carbon imported from non-fruiting and vigorous fruiting spurs (Grappadelli et al., 1994). Early season carbon supply to young apple fruits is critical for fruit cell division, which strongly affects final fruit size (Wünsche and Lakso, 2000a).

In the mid and late season, more assimilates fixed by extension shoot leaves are transported from the shoot to fruits and other branch parts (Wünsche and Lakso, 2000a). This export may be delayed in the season if shoots are shaded (Grappadelli et al., 1994). For a given leaf, transport out of the shoot depends on its position within the shoot and the development stage of the shoot as a whole (Hansen, 1967a). Apple leaves at the base of developing extension shoots exported more than 80% of their assimilates out of the shoot (Hansen, 1967a). In contrast to this, 80% of carbon fixed by top leaves remained in the shoot (Hansen, 1967a). Late in the season, as extension shoots complete their development more assimilates are exported out of the shoot (Hansen, 1967a; Wünsche and Lakso, 2000a). The presence of fruits on the branch also influences the pattern of assimilate distribution. As the fruit grows its sink strength increases and the fruit imports assimilates from distant sources (Hansen, 1967b). Hansen (1969) showed that the leaf/fruit ratio at various points on the branch influences the direction and magnitude of carbon flow. A low leaf/fruit ratio promoted carbon partitioning from extension shoots or spurs without fruit to fruits on other spurs (Hansen, 1969). Black et al. (2000) observed that small fruit size in apple was related to competition between fruits on the same spur. In general, the central or king fruit on the cluster dominated, whereas interspur competition did not affect fruit size (Black et al., 2000). These results suggest that carbon import from distant sources can compensate for interspur competition (Black et al., 2000). Such observations have led to skepticism with respect to the importance of distance in assimilate partitioning between sources and sinks. In another study, carbon import was highest in fruits closest to the source leaf in only 61% of the cases investigated (Hansen, 1969). In most other cases, the highest carbon import was noted in the second closest fruit to the source leaf and in few cases in the third and fourth closest fruit to the source leaf (Hansen, 1969). Such patterns of allocation may be associated with the branch vascular architecture.

## THE ROLE OF THE VASCULAR SYSTEM ARCHITECTURE IN ASSIMILATE PARTITIONING

Branch architecture partly determines the architecture of the vascular system. The vascular system is composed of two types of tissues which generally develop at the same time: the xylem through which water and minerals are transported and the phloem which transports organic materials (De Schepper et al., 2013a). The vascular system connects source organs to various sinks, and thus determines predominant routes for assimilate distribution in the branch (**Figure 1**). Although sink proximity is important in determining the destination of assimilates, instances where a leaf exports carbon predominantly to a remote instead of a closer sink have been reported in apple (Hansen, 1969; Barlow, 1979; Antognozzi et al., 1991). Several authors hypothesized that such observations might be associated with the presence of major vascular connections between the source leaf and a remote sink (Hansen, 1969; Minchin et al., 1997). Indeed, small vascular bundles or traces arising from a leaf can cross one or several nodes to connect with organs positioned at remote locations (Watson and Casper, 1984; Dengler, 2006). Anatomical studies showed that vascular connections are closely related to plant phyllotaxy (Barlow, 1979; Orians et al., 2005; Dengler, 2006). Plant phyllotaxy is represented by a fraction [e.g., 2/5 or 3/8 for apple, (Costes et al., 2006)] of which the numerator and the denominator, respectively, represent the number of turns around the plant axis and the number of internodes between two adjacent leaves/buds superimposed vertically (Watson and Casper, 1984). These vertically aligned leaves/buds are said to belong to the same orthostichy (Watson and Casper, 1984). The denominator of the phyllotactic fraction is either equal to, or a multiple of, the number of main vascular bundles in the stem (Watson and Casper, 1984; Dengler, 2006). For instance, following the 3/8 phyllotaxy in apple, eight main longitudinal bundles traverse the apple branch (Barlow, 1979). Leaves belonging to adjacent orthostichies may be connected to each other, but the most direct vascular connections are between leaves/buds of the same orthostichy (Watson and Casper, 1984). Consequently assimilate flow within the same orthostichy should encounter less resistance than between adjacent orthostichies (Orians et al., 2005). In a study on apple, carbon was primarily exported to successive leaves of the same orthostichy as the source leaf and the lowest export was noted in non-orthostichous leaves positioned opposite to the source leaf (Barlow, 1979). Connectivity between leaves and the reproductive sinks determines major sources for fruit assimilate import (Dražeta et al., 2004).

Modifications of the vascular architecture in response to changes in plant development, stress, management practices etc. may alter major pathways for assimilate distribution. Bud break and the emergence of new branch organs are accompanied by the rapid establishment of new vascular connections (Aloni, 1987). In apple fruits, differentiation of the vascular system in the pedicels during the pre-bloom stage is characterized by the development of small vessels of low conductivity, while large vessels of high conductivity are formed after bloom (Lang and Ryan, 1994; Dražeta et al., 2004). Information on the response of the vascular system to common branch manipulations in apple is rare in the literature. In several other tree species, increases in fruit assimilate import in response to girdling or fruit load treatments have been associated with an increase in the vascular area of the fruit pedicel (Antognozzi et al., 1991; Bustan et al., 1995). Sané et al. (2012) hypothesized that increased fruit assimilate import in the apple inflorescence might be associated with an increased vascularization of the bourse.

## MODELLING AS A TOOL TO UNDERSTAND THE ROLE OF PLANT ARCHITECTURE ON CARBON PARTITIONING

Over the past decades, functional structural plant models (FSPMs) have been proposed to analyze the complex role of plant architecture on carbon production and partitioning. These models, which integrate plant physiological processes and their structural development, are powerful tools to analyze the effect of source or sink number, size, geometry, topology, developmental stage on assimilate production, and partitioning. Han et al. (2012) coupled *MAppleT*, an architectural model of apple with a light model. Light interception in apple was shown to be mostly influenced by internode length and leaf area (Han et al., 2012). More recently Da Silva et al. (2013, 2014) analyzed the influence of apple tree architecture on light interception efficiency. FSPMs containing modules for photosynthesis and light interception have been used to simulate assimilate production at the scale of elementary shoot units or organs (Allen et al., 2005; Massonnet et al., 2008; Baldazzi et al., 2013). Some models introduced feedback inhibition of photosynthesis by assuming that leaf assimilates production was regulated by the amount of carbon it stored (De Schepper and Steppe, 2011). Such models which quantify carbon production of interconnected elementary plant units are suitable for analyzing the role of architecture on the dynamics of carbon transport and partitioning.

Carbon transport in the phloem has been modeled based on Münch pressure flow theory. This theory assumes that phloem carbon is transported by mass flow driven by an osmotically generated pressure gradient between sources and sinks (Ryan and Asao, 2014). A simplified version of the Münch theory where transport is driven by carbon concentration differences between sources and sinks has been used in many FSPMs (Bruchou and Genard, 1999; Allen et al., 2005). However, as pointed out by Minchin and Lacointe (2005) this simplification describes a diffusion process not the mass flow transport known to take place in long distance phloem transport. In many FSPMs carbon transport is constrained by a resistance in the transport path involving the distance between sources and sinks (Bruchou and Genard, 1999; Allen et al., 2005; Génard et al., 2008). This approach was used by Bruchou and Genard (1999) to analyze the effects of fruit positioning on fruit growth within a peach branch. Allen et al. (2005) used an analogy with electric circuitry to describe resistance in the transport path. According to Lang (1979) the phloem may be considered as a series of contiguous short tubes transporting assimilates according to a relay system. Jensen et al. (2012a) proposed to distinguish between resistances in the translocation path and resistances at the source and sink regions. Resistance to carbon flow may be increased by high sap viscosity, for example in the situation of water stress (Hölttä et al., 2009; Sevanto, 2014; Woodruff, 2014) or may be affected by the phloem anatomy (sieve tube length, sieve pore areas; Jensen et al., 2012b; De Schepper et al., 2013a) or the presence of vascular connections between sources and sinks (De Schepper et al., 2013b). Recently Jensen et al. (2012b) proposed a theoretical model to quantify the effects of sieve plates anatomy on phloem transport. Many carbon transport models consider the phloem as a closed conduit, and ignore the exchange of water and sugar taking place between the phloem and the surrounding tissues (Minchin et al., 1993; Allen et al., 2005; De Schepper et al., 2013a). Baldazzi et al. (2013) proposed a model of carbon transport taking into account carbon leakage in the transport path. Considering leakage in a transport model offers the possibility to mechanistically account for the role of storage and remobilization in the regulation of assimilate supply and partitioning (Baldazzi et al., 2013). In several models carbon storage occurs when carbon supply exceeds demand (Lacointe, 2000) or results from competition with other sinks (Balandier et al., 2000; Allen et al., 2005). The relative sink strength of individual sink organs has been used in FSPMs to mechanistically describe carbon partitioning between competing sinks (Escobar-Gutiérrez et al., 1998). Sink demand has also been modeled using thermodynamic equations describing biochemical conversion of sugar (Minchin et al., 1993; Baldazzi et al., 2013).

## FUTURE RESEARCH DIRECTIONS

Though we have shown in this review that many aspects of the role that branch architecture plays in assimilate partitioning have been covered by previous studies, with a large share of studies having been conducted on apple, there are still big holes in the knowledge carpet: at the spur level, the role played by the bourse in redistributing assimilates to fruits is still unclear: as it exhibits a strong secondary growth (swelling) it could be hypothesized that the bourse serves as a local carbon pool, buffering assimilate shortages. However, the temporal dynamics and especially the mechanism of such a function are unknown. Sané et al. (2012) speculated that the key mechanism was secondary vascularization of the bourse.

With respect to the scale of the whole carrier branch three factors affecting carbon partitioning are cited in the literature: the sink force of the fruit, the distance between a sink and the nearest source(s), and, in relation to the previous factor, the direct linking of side branches by common phloem and xylem bundles (vascularization hypothesis). Often, these three factors are not very well distinguished from each other, and authors notably cite the role of phyllotaxy as an explicative hypothesis, without proving its validity. It can equally be asked if this role that phyllotaxy plays in favoring certain branch connections and thus creating carbon partitioning patterns will be lost with age, due to an overlay effect by secondary phloem and xylem formation [effectively, Barlow’s (1979) study seemed to have been valid only for maximally 1-year-old shoots].

Lastly, the direction of partitioning within the branch (proximal or distal) is often unknown, but might be related to the developmental state of its leaves, i.e., the time they turn from sinks into sources, and the way in which they are connected in the phloem network. Hansen (1969) has shown that transport may take place in both directions but this result needs to be validated under controlled conditions and using more of the experimental parameters cited above.

This structured overview could provide the base for knowledge integration in functional-structural plant models of the first-order carrier branch of apple and for further experiment-based research.

## Conflict of Interest Statement

The authors declare that the research was conducted in the absence of any commercial or financial relationships that could be construed as a potential conflict of interest.
